# Perioperative Interventions Based on Fasting Protocols and Carbohydrate Loading in Non-Cardiac Surgery in Older Adults: A Scoping Review

**DOI:** 10.3390/medicina62040756

**Published:** 2026-04-15

**Authors:** Juan David Mejía Lozano, Eduardo Tuta-Quintero, María Camila Bonilla Llanos, María Camila Valencia, Fabián Solano, Andrés Cruz, Nicole Bonilla, Fernando Ríos Barbosa

**Affiliations:** 1 Department of Anesthesiology, School of Medicine, Universidad de La Sabana, Campus del Puente del Común, Km. 7, Autopista Norte de Bogotá, Chía 250001, Colombia; juanmejlo@unisabana.edu.co (J.D.M.L.); mariaboll@unisabana.edu.co (M.C.B.L.); mariavalvar@unisabana.edu.co (M.C.V.); fabiansova@unisabana.edu.co (F.S.); andrescrsi@unisabana.edu.co (A.C.); nicolebosi@unisabana.edu.co (N.B.); 2 Department of Epidemiology, School of Medicine, Universidad de La Sabana, Campus del Puente del Común, Km. 7, Autopista Norte de Bogotá, Chía 250001, Colombia; eduardotuqu@unisabana.edu.co

**Keywords:** perioperative fasting, carbohydrate loading, postoperative delirium, postoperative cognitive dysfunction, older adults, non-cardiac surgery

## Abstract

*Background and Objectives*: Postoperative delirium and postoperative cognitive dysfunction are common complications in older adults undergoing elective non-cardiac surgery, associated with increased morbidity and mortality, functional decline, and prolonged hospital stay. Prolonged preoperative fasting may intensify inflammatory responses and insulin resistance. Preoperative oral carbohydrate loading within ERAS protocols may modulate this response and reduce cognitive risk. *Materials and Methods*: A scoping review was conducted following the methodological recommendations of Arksey and O’Malley, the Joanna Briggs Institute, and PRISMA-ScR. A systematic search was performed in PubMed and Scopus for studies published up to September 2025. Randomized controlled trials and observational studies including adults ≥ 65 years undergoing elective non-cardiac surgery were included if they evaluated fasting modifications or preoperative carbohydrate loading and reported postoperative delirium or cognitive dysfunction. *Results*: A total of eight publications were included: four randomized controlled trials, one prospective cohort study, two cross-sectional studies, and one descriptive/correlational study. Populations included older adults undergoing elective abdominal, orthopedic, colorectal, or hip surgery, as well as hospitalized elderly surgical patients. Interventions included oral carbohydrate loading, assessment of preoperative nutritional status, and enteral versus parenteral nutrition. Only four of the eight included studies directly evaluated neurocognitive outcomes. Postoperative delirium was assessed in three studies, using the Confusion Assessment Method in two studies and the Delirium Rating Scale in one study. Postoperative cognitive dysfunction was evaluated in one study using a Mini-Mental State Examination-based cognitive assessment, while the remaining four studies did not assess neurocognitive outcomes and instead focused on metabolic, inflammatory, or perioperative well-being outcomes. *Conclusions*: Available evidence suggests that perioperative fasting protocols and preoperative carbohydrate loading may influence metabolic and inflammatory responses related to postoperative neurocognitive outcomes in older adults. However, evidence remains limited and heterogeneous. Findings are exploratory and hypothesis-generating, highlighting the need for well-designed trials assessing neurocognitive outcomes in geriatric surgical populations.

## 1. Introduction

Postoperative delirium (POD) and postoperative cognitive dysfunction (POCD) are common complications in older adults undergoing elective non-cardiac surgery, with reported incidences ranging from 10% to 50%, which may increase significantly in patients admitted to intensive care units [1,2,3]. These conditions represent a major clinical challenge due to their association with increased morbidity, mortality, functional decline, and prolonged hospitalization, prompting growing interest in optimizing perioperative brain health [1,2,4].

In the context of global population aging and a sustained increase in surgical procedures among older individuals, there has been increasing emphasis on identifying modifiable perioperative factors that may reduce the risk of cognitive dysfunction [4,5]. Among these, prolonged preoperative fasting has been questioned, as it may exacerbate inflammatory responses, insulin resistance, and physiological instability, particularly in patients with reduced functional reserve. Recent studies also suggest a possible association between extended fasting times and an increased risk of delirium, despite current guidelines allowing clear liquids up to two hours before surgery [1,4].

Preoperative oral carbohydrate (CHO) loading, incorporated into Enhanced Recovery After Surgery (ERAS) protocols, has emerged as a promising strategy to modulate the metabolic response to surgical trauma [6,7]. Available evidence indicates that this intervention may reduce insulin resistance, attenuate inflammatory responses, and improve perioperative well-being and metabolic stability [7,8]. Since systemic inflammation and alterations in cerebral energy metabolism are recognized as key mechanisms in the pathophysiology of POD and POCD, it is plausible that perioperative nutritional strategies may influence these outcomes [9,10].

The evidence linking fasting duration and preoperative CHO administration with neurocognitive outcomes in older adults remains heterogeneous, fragmented, and conceptually dispersed across metabolic, inflammatory, and cognitive domains [1,4,7,8,10,11,12]. In this context, our review aimed to map, synthesize, and describe the available evidence on the association between perioperative fasting protocols and/or preoperative carbohydrate loading and the incidence or severity of POD and POCD in elective non-cardiac surgery among older adults. Rather than presenting new clinical evidence, this scoping review seeks to provide a structured conceptual integration of the existing literature, clarifying current knowledge gaps and identifying directions for future research in this emerging field.

## 2. Methods

A scoping review was conducted following the methodological framework proposed by Arksey and O’Malley [13] and expanded by Levac [14], as well as the methodological guidance of the Joanna Briggs Institute [15] for scoping reviews. Reporting followed the PRISMA Extension for Scoping Reviews (PRISMA-ScR) (Supplementary Table S1) [16]. The review was conducted through five consecutive stages: (1) formulation of the research question, (2) identification of relevant studies, (3) study selection according to predefined criteria, (4) standardized data extraction, and (5) synthesis and presentation of findings.

### 2.1. Research Question

The central question guiding this review was: What evidence is available regarding the association between perioperative fasting protocols and/or preoperative carbohydrate loading and the incidence or severity of POD and/or POCD in older adults undergoing elective non-cardiac surgery?

### 2.2. Objectives

The primary objective of this exploratory review was to map and synthesize existing evidence on the association between perioperative fasting protocols and/or preoperative carbohydrate loading and the incidence or severity of POD and POCD in older adults undergoing elective non-cardiac surgery. Specifically, we aimed to summarize reported interventions, study designs, and outcomes, integrate findings across metabolic, inflammatory, and cognitive pathways, and highlight knowledge gaps to guide future research, emphasizing conceptual understanding rather than new clinical evidence.

### 2.3. Eligibility Criteria

Eligible studies included older adults (≥65 years) undergoing elective non-cardiac surgery; evaluated modified perioperative fasting protocols and/or preoperative carbohydrate or oral solution administration compared with conventional fasting or standard care; and reported postoperative delirium and/or postoperative cognitive dysfunction as outcomes. Randomized controlled trials and observational studies published in English or Spanish were included. Studies exclusively involving cardiac surgery, interventions unrelated to fasting or carbohydrate loading, articles without full-text access, protocols, editorials, and commentaries were excluded.

### 2.4. Information Sources and Search Strategy

Systematic searches were conducted in two electronic databases: PubMed and Scopus. The search strategy was constructed using MeSH terms combined with Boolean operators (Supplementary Table S2). Articles published up to September 2025 were included.

### 2.5. Study Selection Process

Search results were exported in RIS format, and duplicates were removed manually and using a reference manager. Records were then imported into Rayyan [17], where two independent reviewers screened titles and abstracts according to predefined eligibility criteria. Discrepancies or “uncertain” classifications underwent a second review; if disagreement persisted, a third reviewer determined inclusion or exclusion. Full-text assessment followed the same procedure. The entire process was documented using a PRISMA-ScR flow diagram [16].

### 2.6. Data Extraction

Data were extracted independently by two reviewers using a structured template including: author, year and country, study design, population characteristics, study objective, main results, and reported limitations. Findings were synthesized through a descriptive summary of study characteristics and a narrative synthesis organized by study design (clinical trials, observational studies, reviews/meta-analyses) and type of intervention (fasting modification vs. carbohydrate loading), according to categories proposed by Grudniewicz et al. [18].

## 3. Results

**Figure 1:**
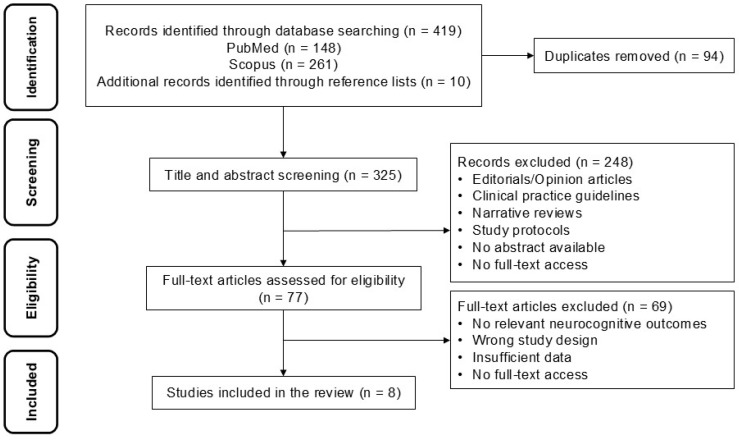
PRISMA flow diagram.

A total of eight publications were included (Figure 1): four randomized controlled trials, one prospective cohort study, two cross-sectional studies, and one descriptive/correlational study. Most studies were conducted in China (*n* = 3), followed by Italy, the United States, India, the United Kingdom, and Iran (*n* = 1 each) (Table 1). Populations included older adults undergoing elective abdominal, orthopedic, colorectal, or hip surgery, as well as hospitalized elderly surgical patients. Interventions included oral carbohydrate loading, assessment of preoperative nutritional status, and enteral versus parenteral nutrition.

Only four of the eight included studies directly evaluated neurocognitive outcomes. POD was assessed in three studies, using the Confusion Assessment Method (CAM) in two studies and the Delirium Rating Scale (DRS) in one study. POCD was evaluated in one study using a Mini-Mental State Examination (MMSE)-based cognitive assessment, while the remaining four studies did not assess neurocognitive outcomes and instead focused on metabolic, inflammatory, or perioperative well-being outcomes.

### 3.1. Clinical Trials

Li et al. [22] evaluated administration of 200 mL of carbohydrates two hours before orthopedic surgery in older adults. The intervention significantly reduced postoperative delirium incidence, IL-6 levels, and glucose levels, while improving thirst, hunger, and overall well-being. Although limited by small sample size (*n* = 80), single-dose CHO administration, and incomplete metabolic assessment, it provides emerging evidence of metabolic and cognitive benefits.

Ghaffari et al. [26] assessed whether oral dextrose improved perioperative well-being in older adults undergoing orthopedic surgery with spinal anesthesia. The intervention reduced thirst, hunger, anxiety, and pain, and decreased an indirect inflammatory marker. Although delirium was not directly assessed, the study supports symptomatic and inflammatory benefits aligned with ERAS principles.

### 3.2. Observational Studies

Denny et al. [21] conducted a cross-sectional study examining preoperative factors and subsyndromal delirium in older adults undergoing joint replacement. Prolonged fasting and recent falls were associated with greater delirium symptom burden. Despite methodological limitations, findings suggest fasting duration may represent a modifiable risk factor.

Viganò et al. [19], in a prospective cohort study, showed that preoperative carbohydrate supplementation reduced plasma glucose, HOMA-IR index, cortisol, and IL-6 in older adults undergoing abdominal surgery. Although cognitive outcomes were not assessed, findings support metabolic modulation through CHO administration.

## 4. Discussion

The findings of this scoping review show that the available evidence regarding the relationship between perioperative fasting protocols, preoperative carbohydrate administration, and neurocognitive outcomes in older adults undergoing elective non-cardiac surgery remains limited yet consistently suggests potential clinical and physiological benefits. The included studies indicate that both oral carbohydrate loading and reduction in traditional fasting may modulate the metabolic response to surgical stress, improve perioperative well-being, and, in some cases, reduce the incidence of postoperative delirium.

This review does not provide new clinical evidence but offers a structured conceptual integration of metabolic, inflammatory, and cognitive outcomes within an emerging and underexplored field. However, heterogeneity in study designs, variability in interventions, and the lack of studies with POD or POCD as primary outcomes reflect a developing field and underscore the need for more robust investigations capable of establishing firm conclusions.

Perioperative carbohydrate loading has been associated with improved patient well-being and modulation of the metabolic response to surgical stress [19,20,21,22,23,24,25,26]. Ricci et al. [27] conducted a network meta-analysis evaluating different preoperative fasting strategies in patients undergoing abdominal surgery and demonstrated that both carbohydrate intake and clear fluid consumption were superior to traditional fasting. The authors reported possible reductions in postoperative nausea and vomiting, as well as improvements in carbohydrate homeostasis, inflammatory response, and length of hospital stay [27]. The cohort study by Marsman et al. [28] showed that flexible fasting was associated with better perceived perioperative well-being, particularly in terms of preoperative thirst, postoperative nausea, and vomiting, highlighting that clear fluid intake up to two hours before surgery was not associated with adverse events.

The available evidence demonstrates methodological heterogeneity and inconsistent results in hard clinical outcomes, leading to a reassessment of international recommendations [19,20,21,22,23,24,25,26]. In this regard, the 2025 update of the ERAS guidelines for colorectal surgery downgraded the strength of recommendation for preoperative carbohydrate loading from strong to weak, based on recent studies that failed to demonstrate consistent clinical benefits across all surgical contexts [1,2,7,29,30,31]. This change emphasizes the need for improved patient stratification, consideration of surgical type and metabolic profile, and the generation of specific evidence in vulnerable populations such as older adults, in whom the effects on neurocognitive and metabolic outcomes may differ from the general surgical population [19,20,21,22,23,24,25,26,29].

Findings from observational studies suggest a possible protective effect on POD mediated by attenuation of the metabolic and inflammatory stress response induced by surgical trauma [19,20,21,22,23,24,25,26]. Surgery is associated with systemic proinflammatory activation characterized by increased cytokines, including interleukin-6 (IL-6), considered a key biomarker in the pathophysiology of delirium and linked to neuronal dysfunction and injury processes [32,33]. In this context, our synthesis identified significant attenuation of metabolic and inflammatory responses during postoperative days 1 and 2, evidenced by reductions in blood glucose levels, insulin resistance, and IL-6 concentrations [19,30,33]. These findings suggest that modulation of surgical stress may indirectly contribute to perioperative neuromodulation and reduction in POD risk, even when this outcome was not directly evaluated [32,33].

Preoperative carbohydrate loading and optimization of fasting protocols may therefore have relevant implications for postoperative neurocognitive outcomes [7,8,9,10,11,12,34,35]. The evidence synthesis presented here may be explained by a shared mechanism involving modulation of metabolic stress, inflammatory response, and perioperative hydration status factors closely linked to the pathophysiology of delirium and postoperative cognitive dysfunction [19,20,21,22,23,24,25,26,36,37,38]. Moreover, the observed association between prolonged fasting, inadequate fluid balance, and increased delirium risk reinforces the notion that traditional fasting may represent a modifiable risk factor for perioperative neurocognitive disorders [19,20,21,22,23,24,25,26,36,37,38]. The consistency of these findings across different surgical contexts and age groups suggests that the impact of prolonged fasting on brain function may transcend chronological age and reflect shared pathophysiological mechanisms [19,20,21,22,23,24,25,26,34,35,38].

Neurocognitive outcomes were often reported as secondary or indirect outcomes in the included studies, and many investigations measured surrogate markers such as inflammatory cytokines or metabolic indicators rather than directly assessing postoperative delirium or cognitive dysfunction with standardized clinical tools [39]. Although systemic inflammation and metabolic dysregulation have a plausible biological basis in the pathophysiology of delirium and cognitive impairment, the evidence from biomarker studies is primarily associative and exploratory, rather than demonstrative of a direct clinical effect on neurocognitive outcomes [39,40]. Furthermore, no single molecule has shown sufficiently robust evidence for clinical application, and marked methodological heterogeneity limits the interpretation of these findings [39,41].

## 5. Limitations

This scoping review has several limitations that should be considered when interpreting its findings. First, consistent with its methodological nature, the objective was to map and synthesize available evidence rather than evaluate effect magnitude or establish causal relationships [13,14,15]; consequently, no formal risk-of-bias assessment or meta-analysis was performed, limiting the ability to draw robust quantitative conclusions [13,14,15]. Second, although the scope of the review was broad, the number of eligible studies was small. Only eight studies met the inclusion criteria, and only one randomized controlled trial directly evaluated postoperative delirium as a primary outcome. Most included studies focused predominantly on metabolic, inflammatory, or perioperative well-being outcomes, with POD and POCD frequently assessed as secondary, exploratory, or non-standardized outcomes [19,20,21,22,23,24,25,26]. Therefore, the evidence directly linking fasting protocols or carbohydrate loading to neurocognitive outcomes remains scarce.

The identified studies were heterogeneous in terms of design, populations, type of surgery, definitions of fasting and nutritional interventions, and instruments used to assess cognitive outcomes, which complicates comparability across studies and limits generalizability [19,20,21,22,23,24,25,26,34,35,38]. Study designs, surgical populations, and types of interventions varied considerably, including oral carbohydrate loading, assessments of nutritional status, and comparisons between enteral and parenteral nutrition. Many studies were not specifically designed for older adults, frequently included small sample sizes, and had limited follow-up. Outcome measures were also inconsistent, and cognitive outcomes were assessed using different instruments or, in some cases, were not evaluated at all, further limiting comparability and generalizability of the findings. These limitations underscore the need for well-designed, adequately powered clinical trials specifically targeting neurocognitive outcomes in geriatric surgical populations [19,20,21,22,23,24,25,26,34,35,38].

Finally, the search strategy was limited to two databases (PubMed and Scopus) and to publications in English and Spanish. Although these are major sources, relevant studies are indexed in other databases, and the grey literature was not systematically searched. These restrictions increase the potential risk of publication bias and may have led to the exclusion of relevant evidence not captured within the selected databases [13,14,15,16].

## 6. Conclusions

Available evidence suggests that perioperative fasting protocols and preoperative carbohydrate loading may influence metabolic and inflammatory responses to surgery, mechanisms that have been hypothesized to be associated with postoperative neurocognitive outcomes such as POD and POCD in older adults. However, the current evidence is limited and heterogeneous, with most studies not primarily designed to evaluate neurocognitive outcomes and often relying on indirect markers.

The findings should be interpreted as exploratory and hypothesis-generating rather than as a basis for clinical recommendations. This review provides a conceptual synthesis linking metabolic, inflammatory, and neurocognitive pathways within an emerging area of research. Future well-designed clinical trials specifically assessing neurocognitive outcomes in geriatric populations are needed to clarify the potential clinical relevance of these perioperative strategies.

## Figures and Tables

**Table 1:** Characteristics of the articles included in the scoping review.

Author, Year, Country [Ref.]	Study Design	Population	Objective	Assessment Tool/Method	Outcome	Neurocognitive Outcomes Assessed	Limitations
Viganò et al., 2012, Italy [19]	Prospective cohort	76 patients undergoing elective abdominal surgery (38 CHO vs. 38 procedure-matched controls)	To evaluate the effect of preoperative oral CHO loading on the metabolic stress response to surgery	Not assessed	The CHO group showed lower postoperative plasma glucose, HOMA-IR index, cortisol, and IL-6 levels compared with controls (*p* < 0.05 for all comparisons). A trend toward lower postoperative infection rates was observed, without statistical significance	Not assessed	Did not assess delirium or POCD; limited sample size; 60% of patients under ERAS protocols
Zhang et al., 2024, China [20]	Cross-sectional study	210 older adults (≥65 years; mean age 75.1 ± 8.8 years; 60.5% male)	To evaluate the association between preoperative nutritional status and POD	Confusion Assessment Method (CAM)	Overall POD incidence: 14.3%. Higher POD risk in patients at risk of malnutrition (OR 2.5; 95% CI) and malnourished (OR 3.4; 95% CI) compared with well-nourished patients. Complication rates: 20.0% (malnourished), 14.3% (at risk), 4.2% (well-nourished)	Primary outcome (POD)	Cross-sectional design; cannot infer causality; single-center study
Denny & Lindseth, 2017, United States [21]	Descriptive correlational	53 older adults (≥65 years; mean age 74 years; 57% women) undergoing joint replacement	To evaluate preoperative risk factors associated with subsyndromal delirium	Delirium Rating Scale (DRS)	68% developed subsyndromal delirium and 17% POD. Longer preoperative fasting was associated with greater delirium symptom burden on postoperative day 3 (r = 0.30; *p* = 0.03)	Secondary outcome	Observational design; single evaluator; potential measurement bias
Li et al., 2025, China [22]	Randomized controlled trial, single-blind	80 older adults undergoing lower limb orthopedic surgery (40 CHO vs. 40 conventional fasting)	To evaluate the effect of preoperative CHO on POD incidence	Confusion Assessment Method (CAM)	POD incidence: 7.5% (CHO) vs. 32.5% (control) (*p* = 0.005). Highest incidence on postoperative day 1 in both groups. Perioperative thirst: 12.5% (CHO) vs. 42.5% (control) (*p* = 0.003)	Primary outcome (POD)	Small sample size; single CHO dose; incomplete metabolic assessment
Gao et al., 2024, China [23]	Cross-sectional study	240 elective surgical patients (120 enteral nutrition [EN] vs. 120 parenteral nutrition [PN])	To compare EN vs. PN on perioperative cognitive function	Mini-Mental State Examination-based perioperative cognitive assessment	POCD incidence: 12% (EN) vs. 20% (PN) (*p* = 0.006). Length of stay: 8.7 ± 1.9 vs. 9.4 ± 2.0 days (*p* < 0.05). 30-day readmission: 10% vs. 15% (*p* < 0.05). Complications: 22% vs. 30% (*p* < 0.05)	Primary outcome (POCD)	Cross-sectional design; cannot establish causality
Kumar et al., 2024, India [24]	Open-label randomized controlled trial	72 patients undergoing elective colorectal surgery (36 CHO vs. 36 control)	To evaluate metabolic, inflammatory, and clinical effects of CHO loading	Not assessed	Significant reduction in insulin resistance in CHO group (*p* = 0.0336). Lower IL-6, CRP, and Glasgow Prognostic Score (*p* < 0.001). Length of stay: 7.0 ± 0.8 vs. 8.6 ± 1.2 days (*p* < 0.001)	Not assessed	Excluded diabetic patients; small sample; did not assess delirium or POCD
Moppett et al., 2014, United Kingdom [25]	Randomized double-blind trial (protocol)	30 patients ≥ 70 years with femoral neck fracture	To evaluate the effect of CHO on insulin resistance and muscle metabolism	Not assessed	Study designed to measure insulin resistance, muscle metabolism, and mobility (Cumulative Ambulation Score); no published results	Not assessed	Protocol without results; did not assess delirium
Ghaffari et al., 2025, Iran [26]	Randomized controlled trial	70 patients undergoing orthopedic surgery under spinal anesthesia	To evaluate the effect of preoperative CHO on perioperative well-being	Not assessed	Significant reduction in thirst, hunger, anxiety, and postoperative pain in CHO group (*p* < 0.05 for all). No increase in postoperative nausea or vomiting	Not assessed	Limited sample size; did not assess cognitive outcomes

Notes: CHO: carbohydrate; POD: postoperative delirium; POCD: postoperative cognitive dysfunction; HOMA-IR: Homeostatic Model Assessment of Insulin Resistance; IL-6: interleukin-6; ERAS: Enhanced Recovery After Surgery; OR: odds ratio; CI: confidence interval; EN: enteral nutrition; PN: parenteral nutrition; CRP: C-reactive protein.

## Data Availability

No new data were created or analyzed in this study.

## Supplementary Materials

The following supporting information can be downloaded at: https://www.mdpi.com/article/10.3390/medicina62040756/s1, Table S1: PRISMA Extension for Scoping Reviews (PRISMA-ScR) 2018 Checklist; Table S2: Search Strategies (search updated 31 December 2025).
